# Radionuclide-Dependent Stimulation of Antitumor Immunity in GD2-Targeted Radiopharmaceutical Therapy Combined with Immune Checkpoint Inhibitors

**DOI:** 10.3390/radiation5040039

**Published:** 2025-12-09

**Authors:** Cynthia Lilieholm, Jen Zaborek, Ohyun Kwon, Adedamola O. Adeniyi, Caroline P. Kerr, Hansel Comas Rojas, Malick Bio Idrissou, Carolina A. Ferreira, Paul A. Clark, Won Jong Jin, Joseph J. Grudzinski, Amy K. Erbe, Eduardo Aluicio-Sarduy, Thines Kanagasundaram, Justin J. Wilson, Jonathan W. Engle, Reinier Hernandez, Bryan Bednarz, Zachary S. Morris, Jamey P. Weichert

**Affiliations:** 1 Department of Radiology, University of Wisconsin School of Medicine and Public Health, University of Wisconsin-Madison, Madison, WI 53705, USA; 2 Department of Pharmaceutical Sciences, University of Wisconsin School of Pharmacy, University of Wisconsin-Madison, Madison, WI 53705, USA; 3 Department of Biostatistics and Medical Informatics, University of Wisconsin School of Medicine and Public Health, University of Wisconsin-Madison, Madison, WI 53705, USA; 4 Department of Medical Physics, University of Wisconsin School of Medicine and Public Health, University of Wisconsin-Madison, Madison, WI 53705, USA; 5 Department of Human Oncology, University of Wisconsin School of Medicine and Public Health, University of Wisconsin-Madison, Madison, WI 53705, USA; 6 Department of Chemistry and Chemical Biology, Cornell University, Ithaca, NY 14853, USA; 7 Department of Chemistry and Biochemistry, University of California Santa Barbara, Santa Barbara, CA 93106, USA

**Keywords:** radiopharmaceutical therapy (RPT), immune checkpoint inhibitors (ICI), GD2-targeted therapy, dinutuximab, dosimetry, combination immunoradiotherapy

## Abstract

Radiopharmaceutical therapy (RPT) offers tumor-selective radiation delivery and represents a promising platform for combination with immune checkpoint inhibitors (ICIs). While prior studies suggest that RPT can stimulate antitumor immunity, synergy with ICIs may depend on radionuclide properties, absorbed dose, and radiation distribution within the tumor microenvironment. This study evaluated how radionuclide selection and dose influence immune stimulation and therapeutic efficacy of GD2-targeted antibody-based RPT combined with ICIs. Dinutuximab, an anti-GD2 monoclonal antibody, was radiolabeled with β^−^-emitters (^90^Y, ^177^Lu) or an α-emitter (^225^Ac). C57Bl6 mice bearing GD2^+^ tumors received 4 or 15 Gy tumor-absorbed doses, determined by individualized dosimetry, with or without dual ICIs (anti-CTLA-4 and anti-PD-L1). In vivo imaging, ex vivo biodistribution, survival, histological, and gene expression analyses were performed to assess therapeutic and immunological outcomes. All radiolabeled constructs demonstrated preferential uptake in GD2^+^ tumors. Combination therapy improved survival in a radionuclide- and dose-dependent manner, with the greatest benefit in the ^225^Ac + ICI group at 15 Gy. Treatment activated type I interferon signaling and increased MHC-I and PD-L1 expression. Notably, ^90^Y reduced regulatory T cells, enhancing CD8^+^/Treg ratios, while ^225^Ac induced robust interferon-driven activation. Radionuclide selection and absorbed dose critically shape immune and therapeutic outcomes of antibody-based RPT combined with ICIs, underscoring the importance of delivery mechanism and dose optimization in combination therapy strategies.

## Simple Summary

Radiopharmaceutical therapy is a type of cancer treatment that delivers radiation directly to tumors using targeted drugs. When combined with drugs called immune checkpoint inhibitors, which help the immune system attack cancer, this approach may improve treatment outcomes. However, not all radioactive drugs behave the same way, and the amount and pattern of radiation delivered inside tumors can change how the immune system responds. In this study, we tested three different radioactive versions of a tumor-targeting antibody in mice with cancer to understand how each affects immune activity and survival. We found that both the type of radioactive particle and the radiation dose play major roles in stimulating helpful immune responses. When paired with immunotherapy, certain combinations led to stronger tumor control. These results may guide the development of safer and more effective treatment strategies that combine targeted radiation with immunotherapy for difficult-to-treat cancers.

## 1. Introduction

Radiopharmaceutical therapy (RPT) is a cancer treatment modality that enables the targeted delivery of ionizing radiation to malignant cells using radiolabeled agents [1]. These radiopharmaceuticals, which consist of a radioactive isotope linked to a targeting vector that recognizes tumor-associated molecular features, serve both diagnostic and therapeutic purposes [2]. Increasing evidence from preclinical models supports the therapeutic potential of combining RPT with immune checkpoint inhibitors (ICIs) to enhance anti-tumor efficacy [3,4]. For instance, NM600 is a small molecule radiotherapeutic that synergizes with checkpoint blockade to produce enhanced tumor regression in murine models [4–8]. This strategy has entered clinical evaluation, including trials investigating ^177^Lu-PSMA-617, an agent approved for metastatic castration-resistant prostate cancer, in combination with ICIs [9–11].

ICIs have transformed cancer treatment by overcoming regulatory mechanisms that suppress T cell activation and function [12]. These agents, which target pathways such as PD-1/PD-L1 and CTLA-4, unleash endogenous anti-tumor immunity and have shown durable responses in a subset of patients across multiple tumor types [12]. However, many malignancies remain resistant to ICI monotherapy or relapse early during treatment [13], highlighting the need for complementary approaches that stimulate the tumor immune microenvironment.

Monoclonal antibodies serve as effective targeting vectors in RPT by delivering cytotoxic radiation to antigen-expressing tumor cells, thus sparing normal tissues [14]. When combined with ICIs, antibody-based RPT offers the dual advantage of direct tumor cytotoxicity and potential immunogenic modulation [1,14]. This strategy is particularly compelling in the context of GD2, a disialoganglioside that is overexpressed in neuroblastoma, melanoma, and certain sarcomas but exhibits minimal expression in normal tissues [15,16]. Dinutuximab, an anti-GD2 monoclonal antibody approved for high-risk neuroblastoma, mediates tumor cell killing via immune effector mechanisms and is currently under clinical evaluation for other GD2-positive tumors [16,17]. However, due to their relatively large molecular size, monoclonal antibodies exhibit limited tissue penetration and heterogeneous intratumoral distribution, which may restrict therapeutic efficacy, particularly in poorly vascularized or densely cellular tumor regions [18].

Previous studies have shown that external beam radiation therapy (EBRT) can enhance ICI efficacy by promoting immune cell infiltration and altering the tumor microenvironment [19,20]. In some cases, EBRT has been observed to induce abscopal effects, wherein immune-mediated tumor regression occurs at sites distant from the irradiated field, likely due to systemic activation of antitumor immunity [20,21]. However, unlike RPT, EBRT is inherently limited in its capacity to irradiate all tumor sites simultaneously in patients with widespread metastatic disease. This spatial limitation may constrain its ability to stimulate a systemic immune response [4]. In contrast, RPT enables systemic delivery of targeted radiation to both visible and occult tumor lesions [22], making it particularly well-suited for combination with immunotherapy. Indeed, targeted RPT has shown encouraging synergy with ICIs in preclinical and early-phase clinical studies [3,9,10,23]. Radiation itself is known to stimulate antitumor immunity through mechanisms such as immunogenic cell death and activation of dendritic cells [24]. Importantly, heterogeneous radiation dose distribution within tumors may further amplify immune responses and facilitate checkpoint blockade sensitivity. For instance, brachytherapy, which delivers high-dose radiation locally while minimizing exposure to surrounding tissues, can influence the immune response and tumor microenvironment [25,26]. Low-dose radiation has also been shown to reduce cancer-associated fibroblasts and enhance T-cell infiltration [27]. These immunomodulatory effects support the rationale for exploring low- and intermediate-dose RPT in combination with immunotherapy [4,7,8,27].

## 2. Materials and Methods

### 2.1. Cell Lines and Culture

The murine melanoma B78-D14 (B78) cell line, derived from B16 melanoma as previously described, was obtained from Dr. Ralph Reisfeld (Scripps Research Institute; La Jolla, CA, USA) in 2002 [28]. The B16F10(B16) melanoma cell line, syngeneic to C57BL/6 mice, was originally obtained from Dr. William Ershler (University of Wisconsin–Madison; Madison, WI, USA) [29]. B78 cells have functional GD2/GD3 synthase and express the disialoganglioside GD2 whereas the B16 cell line lacks GD2 expression [28,30,31]. The 9464D cell line was originally obtained from Dr. Jon Wigginton (National Cancer Institute; Bethesda, MD, USA) and was developed by Dr. William Weiss (University of California, San Francisco; San Francisco, CA, USA) from spontaneous neuroblastoma tumors arising in TH-MYCN transgenic mice on a C57BL/6 background [32,33]. The neuroblastoma 9464DGD2 cell line, derived from 9464D neuroblastoma as previously described, was obtained from Dr. Paul Sondel (University of Wisconsin–Madison) [32]. 9464D-GD2 cells have functional GD2 and GD3 synthases and were selected for interferon-γ inducible MHC-I expression [34]. All cells were grown in a humidified incubator at 37°C with 5% CO_2_. B78 cells were grown in RPMI-1640 supplemented with 10% FBS, 100U/mL penicillin, and 100 μg/mL streptomycin. B16 and 9464D cells were grown in DMEM supplemented with 10% FBS, 100U/mL penicillin, and 100 μg/mL streptomycin. 9464D-GD2 cells were additionally supplemented with 1× MEM non-essential amino acids and antibiotics (puromycin at 6 μg/mL and blasticidin at 7.5 μg/mL) to maintain expression of GD2 and GD3 synthases. Cell line authentication was performed according to ATCC guidelines based on morphology and growth characteristics. Mycoplasma testing was routinely performed and was confirmed negative using MycoStrip (InvivoGen; San Diego, CA, USA).

### 2.2. Murine Tumor Models

All mouse studies were approved by the Institutional Animal Care and Use Committee at the University of Wisconsin–Madison (protocol: M005853) and conducted in accordance with the Research Animal Resource Center guidelines. C57BL/6 mice were purchased from Taconic at 8–10 weeks of age for melanoma tumor models, and from JAX at 5–7 weeks of age for neuroblastoma tumor models. All mice were stabilized for seven days before the initiation of research studies. Both male and female mice were included in therapy studies, while only female mice were used for imaging and correlative studies.

#### 2.2.1. Imaging Studies

B78 tumors were engrafted by subcutaneous flank injection of 2 × 10^6^ tumor cells on the right flank five weeks before imaging (tumor size ~150–200 mm^3^), and B16 tumors were engrafted by subcutaneous injection of 1 × 10^6^ tumor cells on the left flank of the same mice (n = 4) two weeks before imaging (tumor size ~200–300 mm^3^). Tumor size was determined using calipers and volume approximated as (length × width^2^)/2. 9464D-GD2 tumors were engrafted by subcutaneous flank injection of 2 × 10^6^ tumor cells on the right flank three weeks before imaging (~100–150 mm^3^), and 9464D tumors were engrafted by subcutaneous injection of 2 × 10^6^ tumor cells on the left flank two weeks prior (~150–200 mm^3^) for PET/CT, SPECT/CT, and ex vivo biodistribution gamma counting. B78 tumors were engrafted for alpha camera imaging five weeks prior (~150–200 mm^3^) by subcutaneous injection of 2 × 10^6^ tumor cells on the right flank.

#### 2.2.2. Therapy Studies

B78 tumors were engrafted by subcutaneous injection of 2 × 10^6^ tumor cells. Mice were randomized immediately before treatment based on tumor size. Only mice with palpable flank tumors were included in the study at four weeks (tumor volume ~50–100 mm^3^). The day of RPT was defined as “day 1” of treatment. Anti-murine CTLA-4 (IgG2c, clone 9D9, NeoClone; Madison, WI, USA) and anti-murine PD-L1 (IgG2b, clone 10F.9G2, BioXCell; Lebanon, NH, USA), 100 μg each, were administered by intraperitoneal injection on days 4, 7, and 10. Mice were euthanized when tumor size exceeded 2000 mm^3^ in volume or when recommended by an independent animal health monitor due to morbidity or moribund behavior. Due to institutional radiation safety protocols and the use of multiple radioactive isotopes, all investigators were aware of mouse treatment groups. Delays in radioisotope shipments may have caused variation in tumor size on day 1. Therapy experiments were repeated in duplicate. The number of animals per group and average tumor size at treatment initiation are indicated in figure legends.

#### 2.2.3. Toxicity and Immunoprofiling Studies

B78 tumors were engrafted by subcutaneous injection of 2 × 10^6^ tumor cells on both left and right flanks. At five weeks (~150–200 mm^3^), mice were randomized by tumor size. Treatment groups included 4 Gy, 4 Gy + ICI, 15 Gy, 15 Gy + ICI, and a cold dinutuximab + ICI control group. Mice were euthanized via CO_2_ asphyxiation on D0 (n = 5; naïve control) and on days 4, 7, 14, and 21 (n = 4 per timepoint per treatment). Blood was collected for toxicity assessments and flow cytometry. Tumors from both flanks were either processed for flow cytometry or flash frozen in LN_2_ and stored until decay allowed for gene expression analysis.

### 2.3. Radiopharmaceuticals

^90^Y was purchased as ^90^YCl_3_ from Eckert and Ziegler (Valencia, CA, USA). ^177^Lu was purchased as ^177^LuCl_3_ from Shine Medical (Janesville, WI, USA) or Oak Ridge National Laboratory (Oak Ridge, TN, USA). ^225^Ac was obtained as solid ^225^Ac(NO_3_)_3_ from Oak Ridge National Laboratory. ^89^Zr was produced in a GE PETtrace cyclotron (University of Wisconsin–Madison) by irradiating natural yttrium foils (250 μm, 99.9%) with 13.8 MeV protons, as previously described [35]. Dinutuximab (Unituxin^®^, United Therapeutics; Silver Spring, MD, USA) was reconstituted in sterile phosphate-buffered saline (PBS) and purified using PD-10 desalting with PBS as the mobile phase. Chelators were conjugated to dinutuximab via exposed lysine residues using established protocols [36,37]. For ^89^Zr, p-SCN-Deferoxamine (DFO, Macrocyclics; Plano, TX, USA) was used; for ^90^Y and ^177^Lu, p-SCN-Bn-CHX-A^′′^-DTPA (DTPA, Macrocyclics); for ^225^Ac, H_2_macropa-NCS (macropa) provided by Dr. Justin Wilson (Cornell University; Ithaca, NY, USA). As an actinide, ^225^Ac exhibits limited stability when coordinated with conventional chelators such as DOTA, often resulting in suboptimal radiolabeling efficiency and in vivo stability. In contrast, the novel chelator macropa has demonstrated markedly improved performance, characterized by higher radiochemical yield, radiochemical purity, specific activity, and serum stability, as well as enhanced tumor uptake in preclinical models [38–41]. DFO and DTPA were dissolved in anhydrous DMSO and mixed with dinutuximab at a 5:1 molar ratio; pH adjusted to ~8.5 using 0.1 M Na_2_CO_3_. Macropa was used at a 15:1 molar ratio and incubated overnight at 4°C. DFO and DTPA reactions were incubated at room temperature for 1~4h. All conjugates were purified via PD-10 columns and concentrated using Amicon Ultra centrifugal filter 30K MWCO (Merck; Darmstadt, Germany) at 4000 g for 15 min, then stored at 4°C. For radiolabeling, 1M HEPES buffer was added to ^89^Zr for pH 7.5, 0.1M NaOAc buffer was added to ^90^Y for pH 5.5, 1M NaOAc buffer was added to ^177^Lu for pH 5.5, and 0.1M HEPES buffer with 0.4% *v*/*v* Tween-20 was added to ^225^Ac for pH 5.5. The neutralized radioisotopes were added to the antibody-chelator conjugate at a ratio of 0.1 mg of DFO-dinutuximab or DTPA-dinutuximab per 37 MBq of ^89^Zr, ^90^Y, and ^177^Lu. For ^225^Ac, 5 μg of macropa-dinutuximab conjugate was added per 37 kBq. Final volume was adjusted to 500 μL with buffer and incubated at 37°C for 1 h. Final products were purified by PD-10 columns and diluted in sterile PBS. For dose consistency, additional unlabeled dinutuximab was added to the low dose (4 Gy) formulations to match antibody content of high dose (15 Gy) formulations based on the apparent molar activities. Radiolabeling yield and purity were assessed via instant thin-layer chromatography using Perkin Elmer silica paper (iTLC-SA) and pH 8.0 50mM EDTA as solvent. Labeled antibody remained at the origin; free radiometals migrated with solvent. A Perkin Elmer Cyclone Plus image reader was used to analyze the chromatograms for quantifying labeling efficiency. For radiolabeling efficiency, n = 4 was performed for all radiolabeled isotopes.

### 2.4. PET/CT Imaging

Mice (n = 4) bearing B78 and B16 (~150 mm^3^) or 9464D and 9464D:GD2^+^ tumors (~150 mm^3^) were injected intravenously via tail vein with 9.25 MBq of ^89^Zr-dinutuximab. Imaging was performed at 3, 24, 72, and 168 h post injection using an Inveon microPET/CT scanner (Siemens Medical Solutions, Knoxville, TN, USA). Mice were anesthetized with 2% isoflurane and placed prone on the scanner bed. Sequential CT (80 kVp; 1000 mAs; 220 angles) and static PET scans (80 million coincidence events; time window: 3.432 ns; energy window: 350–650 keV) were collected. A three-dimensional ordered subset expectation maximization algorithm was used to reconstruct the PET images. These were then fused with corresponding CT images for attenuation correction and anatomical referencing. Tumors were contoured for region-of-interest analysis to quantify ^89^Zr-dinutuximab uptake as %IA/g (mean ± SEM) and used for dosimetry.

### 2.5. SPECT/CT Imaging

Mice (n = 4) were injected intravenously with 18.5 MBq of ^177^Lu-dinutuximab and imaged using a MILabs U-SPECT6/CTUhr system (Houten, The Netherlands) at 24, 72, 168, and 336 h post-injection. Mice were anesthetized with 2% isoflurane and placed prone on a 4-mice multi bed. CT scans (10 min) were acquired for anatomical reference and attenuation correction and fused with the SPECT scans (45 min). Image reconstruction used a similarity-regulated ordered-subset expectation maximization (SROSEM) algorithm. Tumors were contoured for volumes-of-interest analysis and uptake quantified as %IA/g (mean ± SEM), used for dosimetry calculations.

### 2.6. Alpha Camera Imaging

Mice (n = 3) were injected via tail vein with 9.25 MBq ^90^Y-dinutuximab, 18.5 MBq ^177^Lu-dinutuximab, or 7.4 kBq ^225^Ac-dinutuximab. Mice were euthanized via CO_2_ at 72 h post-injection, and tumors harvested. Tumors were bisected and embedded in Tissue-Tek OCT compound, frozen in −80°C, and sectioned to 10 μm using a CM1950 cryostat (Leica; Deer Park, IL, USA). After sectioning, slides were placed against the iQID (QScint; Tucson, AZ, USA) detector window separated from the input window by a thin sheet of mylar (Ludlum Measurements 01–5859; Sweetwater, TX, USA) and the scintillator of choice depending on the radioisotope being scanned. For ^225^Ac-dinutuximab, a ZnS: Ag detector was used (Eljen Technology EJ-440; Sweetwater, TX, USA) and for ^90^Y- and ^177^Lu-dinutuximab, an image intensifying screen meant to detect low-and-medium-energy beta particles was used (Carestream BioMax TranScreen LE; Rochester, NY, USA). An in-house slide holder was used for experiments in order to fix scan bed position of the glass slides for subsequent image registration purposes between other imaging modalities. One scan was performed per slide and all lasted in duration depending on isotope; 1 h duration each for all ^177^Lu-dinutuximab scans, one 2.6 h and two 1 h scan durations for ^90^Y-dinutuximab scans, and one 1.9 h, one 4 h and one 1 h scan for ^225^Ac-dinutuximab.

### 2.7. Ex Vivo Biodistribution

Mice were injected intravenously with 7.4 kBq ^225^Ac-dinutuximab and euthanized at 3, 24, 72, and 168 h post-injection (n = 3/timepoint). Organs were harvested, weighed, and stored at 4°C to reach secular equilibrium with ^213^Bi. Samples were analyzed using a Perkin Elmer Wizard2 (Westham, MA, USA) or Hidex AMG (Turku, Finland) gamma counter, with decay correction to calculate %IA/g.

### 2.8. Dosimetry Calculations

**Figure 1.**
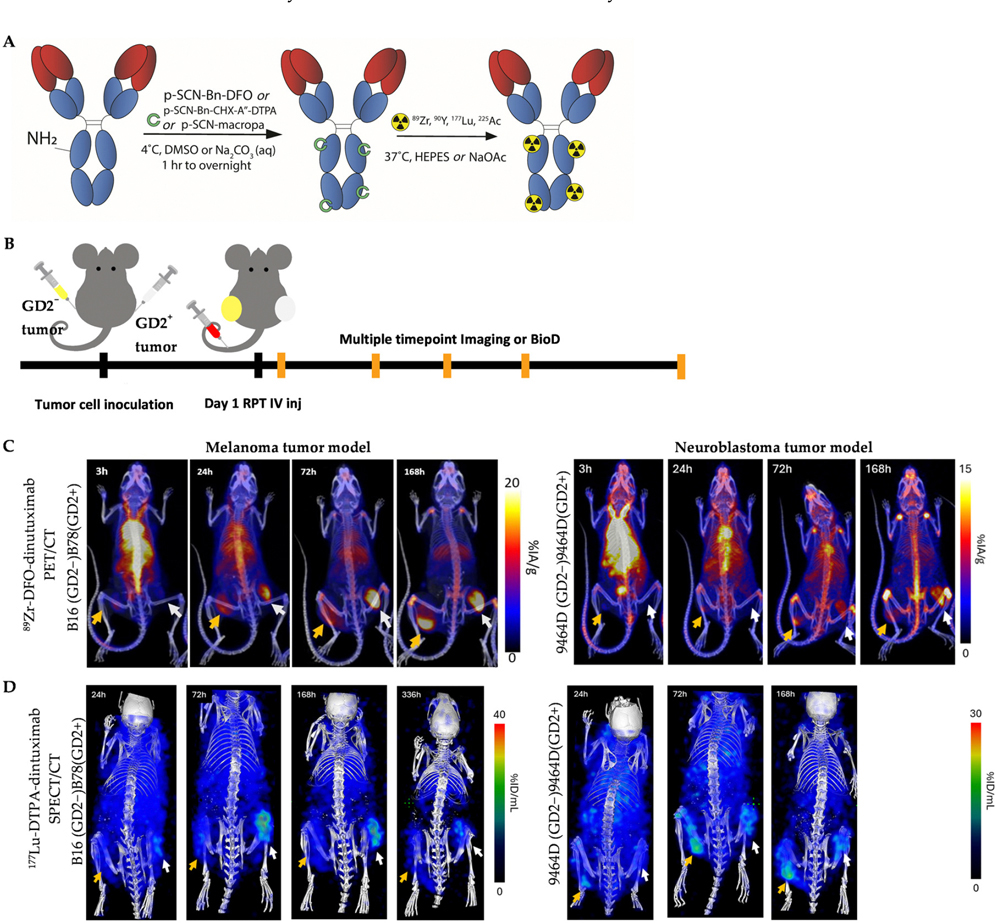

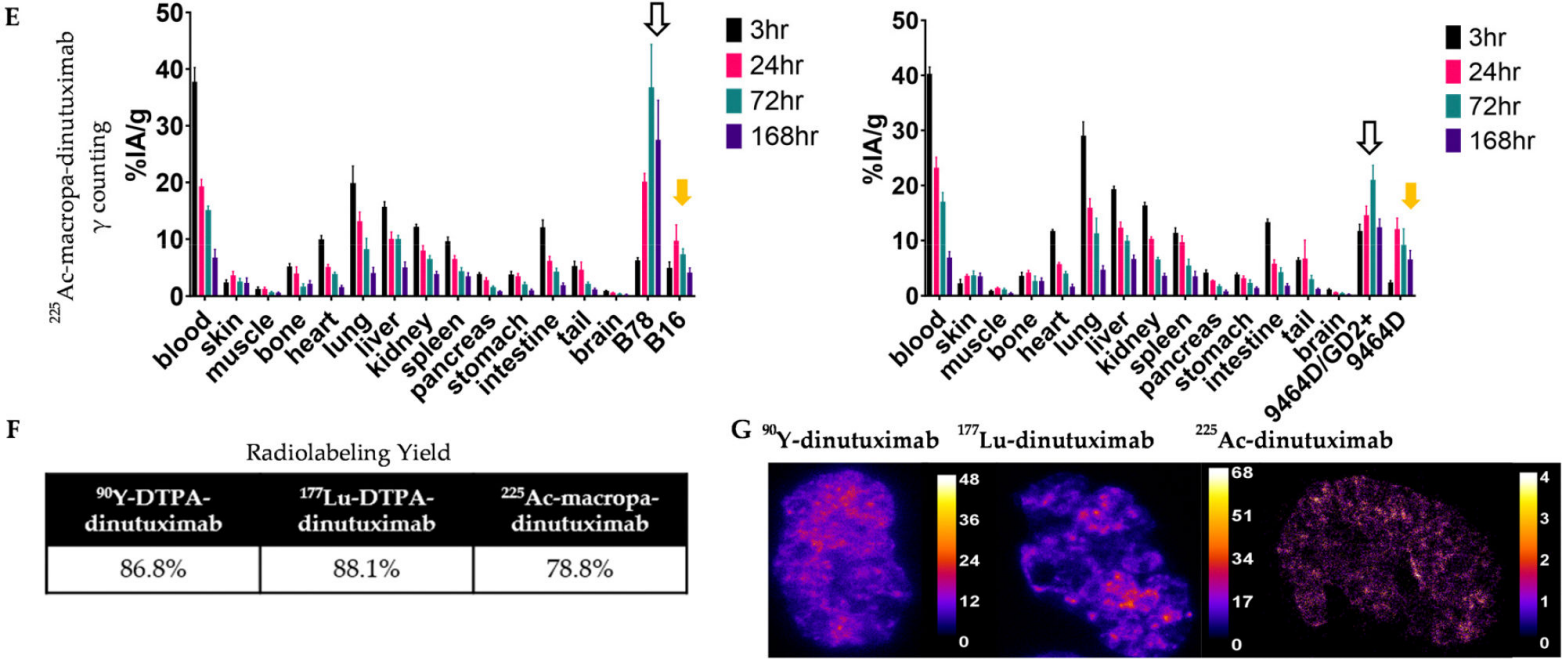
Imaging, uptake, and distribution of ^89^Zr-DFO-dinutuximab, ^177^Lu-DTPA-dinutuximab, and ^225^Ac-macropa-dinutuximab. (**A**) Schematic representation of the radiolabeling process. The chimeric anti-GD2 monoclonal antibody dinutuximab was conjugated with the appropriate chelators labeled in green-deferoxamine (DFO) for ^89^Zr, diethylenetriaminepentaacetic acid (DTPA) for ^177^Lu, and macropa for ^225^Ac-via thiourea bond. Radiolabeling was performed under mild conditions (pH 5.5–7.0) for 1 h at 37°C. (**B**) Bilateral tumor-bearing C57BL/6 mice were used, with syngeneic GD2-negative (yellow arrow) and GD2-positive (white arrow) B16/B78 melanoma or 9464D/9464D:GD2^+^ neuroblastoma tumors implanted on the left and right flanks. Tumors were allowed to reach ~180 mm^3^ before radiopharmaceutical administration. (**C**) Longitudinal PET/CT imaging of mice injected with ^89^Zr-DFO-dinutuximab (∼9.25 MBq) was performed at 4, 24, 72, and 168 h post-injection. (**D**) Serial SPECT/CT imaging of ^177^Lu-DTPA-dinutuximab (∼18.5 MBq) was performed at 24, 72, 168, and 336 h post-injection. (**E**) For ^225^Ac-macropa-dinutuximab (∼7.4 kBq), quantitative ex vivo biodistribution was conducted at 4, 24, 72, and 168 h post-injection. Tissues were harvested, weighed, and radioactivity was measured via gamma counting of ^213^Bi emissions to estimate ^225^Ac decay products. (**F**) Radiochemical yields (n = 4) for each construct are summarized in a table format, as measured by iTLC. (**G**) Autoradiography of ^90^Y-, ^177^Lu-, and ^225^Ac-dinutuximab treated tumor sections. Cryosections were obtained at 20 μm thickness, exposed to phosphor screens, and imaged with an iQID scanner (QScint; Tucson, AZ, USA).

To estimate the mean absorbed dose of ^225^Ac-dinutuximab, organs of interest were collected from each mouse group at 3, 24, 72, and 168 h timepoints. Ex vivo biodistribution was measured at each timepoint as the percentage of injected dose per gram of tissue (%ID/g) using a gamma counter (PerkinElmer; Waltham, MA, USA). Allometric scaling was first performed to estimate the intact organ mass with respect to body mass, assuming 20 g for all C57BL/6 mice. Cumulative activity (MBq-s/MBq_injected) in each organ was calculated by trapezoidal integration of the time activity curves, assuming physical decay only beyond the final timepoint. Absorbed dose to each organ was determined by multiplying the cumulative activity by the corresponding MIRD S-value (mGy/MBq-s) dose factors. S-values were derived assuming each organ as a liquid water sphere of equivalent mass receiving only self-dose. The total absorbed dose for each organ accounted for the full decay chain of ^225^Ac, considering negligible redistribution of the progenies. The redistribution and off-target accumulation of ^225^Ac daughter radionuclides remain an active area of investigation. Although we are not aware of a study that precisely matches our pharmaceutical construct and tumor model, a recent study using ^225^Ac-labeled PSMA ligands reported that the progeny of ^225^Ac-PSMA I&T is trapped in tumor tissue [42]. We acknowledge that some daughter redistribution (e.g., ^213^Bi) has been observed in other models, with accumulation reported in the kidneys, liver, or salivary glands via systemic circulation [43]. However, in our ex vivo biodistribution study measuring &IA/g (Figure 1E), we did not observe elevated uptake in the kidneys or liver, suggesting limited redistribution in our specific model. PET/CT- and SPET/CT-based dosimetry followed published methods [6,44,45]. %IA/g extrapolation and standard mouse models were used to convert cumulative activity to absorbed dose per injected activity (Gy/MBq). Dose contributions from surrounding organs were also included in calculations. For all absorbed dose calculations, we accounted for both the biological and physical half-lives of the radiopharmaceutical agents. Time-activity curves (TAC) were constructed using the serial measurements obtained either from imaging data or gamma counter-based quantification, depending on the experimental group. These data were used to estimate time-integrated activity coefficients for accurate dosimetry.

### 2.9. Toxicity Assessments

A comprehensive metabolic panel (CMP) and complete blood count (CBC) analyses were conducted for mice included in toxicity and immunoprofiling studies. All mice had 700 μL of blood collected via intracardiac puncture. CBC analysis was performed using whole blood collected in EDTA tubes and analyzed on a VetScan HM5 hematology analyzer (Abaxis; Union City, CA, USA). Serum was separated by centrifugation at 4000 rpm for 10 min, then analyzed using a VetScan VS2 analyzer (Abaxis). Samples not analyzed on the same day were stored at −20°C and thawed on ice prior to analysis.

### 2.10. Radiopharmaceutical Therapy (RPT)

^90^Y-, ^177^Lu-, or ^225^Ac-dinutuximab therapy was administered via intravenous tail vein injection on treatment day 1 for all animal studies. Table 1 shows the injected activities corresponding to the prescribed tumor absorbed doses.

### 2.11. Gene Expression Analysis

Tumors were harvested and homogenized in Trizol using a Bead Mill Homogenizer Bead Ruptor Elite (Omni International; Kennesaw, GA, USA). Total RNA was extracted using the RNeasy Mini Kit (QIAGEN; Germantown, MD, USA) per the manufacturer’s protocol. Complementary DNA (cDNA) was synthesized using the QuantiTect Reverse Transcription Kit (QIAGEN). Quantitative PCR (qRT-PCR) was performed using TaqMan Fast Advanced qPCR Master Mix (ThermoFisher; Waltham, MA, USA). Thermal cycling conditions on the QuantStudio 6 (Applied Biosystems; Foster City, CA, USA) were as follows: UDG activation at 50°C for 2 min, Dual-Lock DNA polymerase activation stage at 95°C for 2 min, then 40 PCR cycles—denaturation at 95°C for 1 s and annealing/extension at 60°C for 20 s. Ct values were exported to Excel, and fold changes normalized to untreated controls were calculated using the ΔΔCt method. *Hprt* served as endogenous controls. A complete list of TaqMan probes is provided in Table S1. All qPCR experiments were performed in duplicate and presented as aggregate data.

### 2.12. Flow Cytometry

Flow cytometry was performed as previously described [46]. UltraComp eBeads fluorescent beads (Invitrogen; Carlsbad, CA, USA) were used for compensation, and fluorescence minus one (FMO) controls were used for gating. Rainbow beads (Spherotech; Lake forest, IL, USA) were used to match voltages to D0 settings. For in vivo analyses, tumors, spleens, and tumor draining lymph nodes were harvested and dissociated using 70 μm Falcon cell strainers (Corning; Corning, NY, USA). Blood was collected via intracardiac puncture. Spleens and blood were treated with RBC lysis buffer (Biolegend; San Diego, CA, USA) and washed with PBS prior to staining. To prevent non-specific binding, cells were incubated with CD16/32 antibody (BioLegend). Live dead staining was performed using Ghost Red Dye 780 (Tonbo Biosciences; San Diego, CA, USA) per manufacturer’s instructions. Afterward, single-cell suspensions were labeled with surface antibodies at 4°C for 30 min then washed three times with flow buffer (2% FBS + 2 mM EDTA in PBS). For intracellular staining, cells were fixed and stained for internal markers with Cytofix/Cytoperm permeabilization solution (BD Biosciences; Franklin Lakes, NJ, USA) according to manufacturer’s instructions. Flow cytometry was conducted on a ThermoFisher Attune NxT Flow Cytometer. Data were analyzed using FlowJo Software v10. A complete list of antibodies, clones, and fluorophores is provided in Table S2. All experiments were repeated in duplicate and presented as aggregate data.

### 2.13. Statistical Analysis

All statistical analyses were conducted using Graphpad Prism 10 and R 4.4.2. Two-way ANOVA or multiple comparison tests using Tukey’s honestly significant difference (HSD) test were applied to assess group differences in gene expression and flow cytometry data. For statistical analysis of microdosimetry results, separate linear models for each radionuclide were fit to estimate the effect of tumor type (melanoma vs. neuroblastoma) on required dose, adjusting for volume and prescription level. Arithmetic models used the raw required dose (μCi) as the outcome and geometric models used log-transformed required dose to estimate percent differences. For tumor growth analysis, all available data was used. For tumor growth and survival analysis, all available data was used (Figure S2). For experiments on varied radionuclides and ICI treatment, linear mixed models after log base 10 transformation of tumor volume were used with fixed effects covariates of radionuclide, time in weeks, and their interaction. Mouse ID was included as a random intercept. To compare radiation level, ICI, and antiGD2 treatment groups within each radionuclide, linear mixed models were again used, retaining log-transformed tumor volumes as the outcome and mouse ID as a random effect. The fixed effects were treatment group, time in weeks, and the interaction between treatment and time. Cox proportional hazards models were used to estimate differences in overall survival. Models were fit for each treatment group to estimate the differences between radionuclides. Then, additional models were fit for each radionuclide to estimate the differences by treatment group. All data are reported as mean ± standard error of the mean (SEM) unless otherwise noted. For tumor growth and survival graphs, *, *p* < 0.03; **, *p* < 0.002; and ***, *p* < 0.001. For gene expression analysis and flow cytometry graphs, *, *p* < 0.03; **, *p* < 0.0021; ***, *p* < 0.0002; and ****, *p* < 0.0001.

## 3. Results

### 3.1. Imaging, Uptake, and Distribution of ^89^Zr-DFO-, ^177^Lu-DTPA-, and ^225^Ac-Macropa-Dinutuximab

The primary aim of this study was to evaluate the immunostimulatory potential of antibody-based RPT using different radionuclides in combination with ICIs. As a foundational step toward this goal, we first characterized the in vivo imaging, uptake, and distribution profiles of ^89^Zr-, ^177^Lu-, and ^225^Ac-dinutuximab in syngeneic murine models. Dinutuximab was conjugated with appropriate chelators and radiolabeled with each radionuclide (Figure 1A). Bilateral tumor models were established, with GD2-negative and GD2-positive melanoma or neuroblastoma tumors implanted on opposite flanks of each mouse (Figure 1B).

^89^Zr-labeled dinutuximab was used as a PET surrogate for ^90^Y-dinutuximab, consistent with its conventional role in theranostic applications [37,47]. The half-life of ^89^Zr (3.3 days) aligns more closely with ^90^Y (2.67 days) than other isotopes such as ^86^Y (0.618 days) [48,49]. Serial PET/CT imaging revealed preferential uptake of ^89^Zr-dinutuximab in GD2-positive tumors, consistent with target-specific binding. Similarly, ^177^Lu-dinutuximab demonstrated selective accumulation in GD2-positive tumors on SPECT/CT (Figure 1C,D) which was corroborated by ex vivo biodistribution at the final timepoint (Figure S1A). Both ^89^Zr- and ^177^Lu-dinutuximab showed progressive clearance from non-target tissues and sustained retention in tumors up to seven days post-injection.

Due to imaging limitations of alpha-emitters, ex vivo biodistribution was used to evaluate ^225^Ac-dinutuximab. Temporal biodistribution profiles indicatd consistent and prolonged accumulation in GD2-positive tumor tissue (Figure 1E). Radiochemical yields for all conjugates exceeded 80% as confirmed by iTLC (Figure 1F). Despite the limited penetration depth of monoclonal antibodies and the short path length of alpha particles, autoradiographic analysis suggests ^225^Ac-dinutuximab is distributed intratumorally (Figure 1G).

### 3.2. Dosimetry Highlights the Need for Absorbed Dose-Based RPT Evaluation

**Figure 2.**
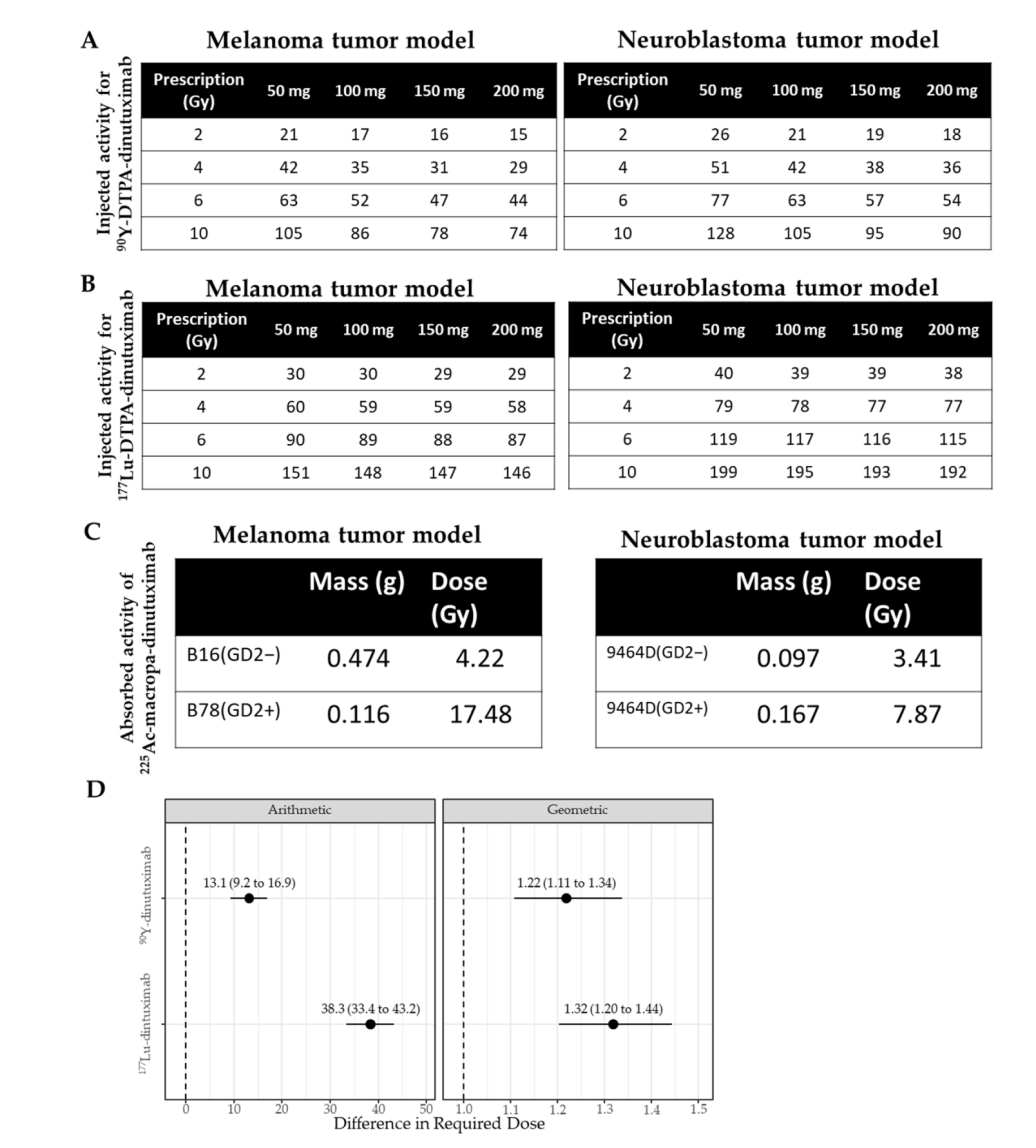
Different microdosimetry results based on the same vector, radionuclide and chelator but in different tumor models. (**A**) Based on the absorbed dose estimates for the tumor with ^89^Zr-dinutuximab, a table was created to inform approximations for injected activity of ^90^Y-dinutuximab needed to deliver a prescribed dose to a tumor of different masses. (**B**) The required amount of injected activity (μCi) of ^177^Lu-dinutuximab to achieve a prescription absorbed dose (Gy) in a corresponding tumor mass (mg). (**C**) B16/B78 tumors received an absorbed dose of 21.1 and 87.4 Gy/μCi, and 9464D/9464D:GD2+ tumors received an absorbed dose of 17.1 and 39.3 Gy/μCi, respectively. For an injected activity of 0.2 μCi, the absorbed doses to the B16/B78 were 4.22 and 17.5 Gy and 9464D/9464D:GD2+ tumors were 3.41 and 7.87 Gy, respectively. (**D**) Statistical difference between required dose (μCi) in melanoma and neuroblastoma tumors under arithmetic and geometric considerations for ^90^Y- and ^177^Lu-dinutuximab. Dotted lines indicate the baseline.

While injected activity remains the clinical standard for RPT dosing, it fails to account for inter-model variability in tumor uptake and radiation distribution [50–53]. To better reflect biological impact, voxel-based absorbed dose estimates were derived using longitudinal PET/CT or SPECT/CT data and a Monte Carlo-based dosimetry platform [45] (Figure 2A,B). Absorbed dose calculations revealed substantial differences between models, even when the same radiolabeled construct was used. For ^225^Ac-dinutuximab, tumor dose was estimated via its ^213^Bi daughter (t_½_ = 45.6 min), which enabled higher-resolution microscale dose modeling (Figure 2C). Overall, neuroblastoma tumors had a higher injected activity (μCi) required than melanoma tumors to achieve any equivalent tumor deposited dose (Figure 2D). For ^90^Y-dinutuximab, this was 22% greater (confidence interval 11–34%, *p* < 0.001) and for ^177^Lu-dinutuximab, this was 32% greater (20–40%, *p* < 0.001).

### 3.3. RPT and ICI Synergy Is Both Dose- and Radionuclide-Dependent

**Figure 3.**
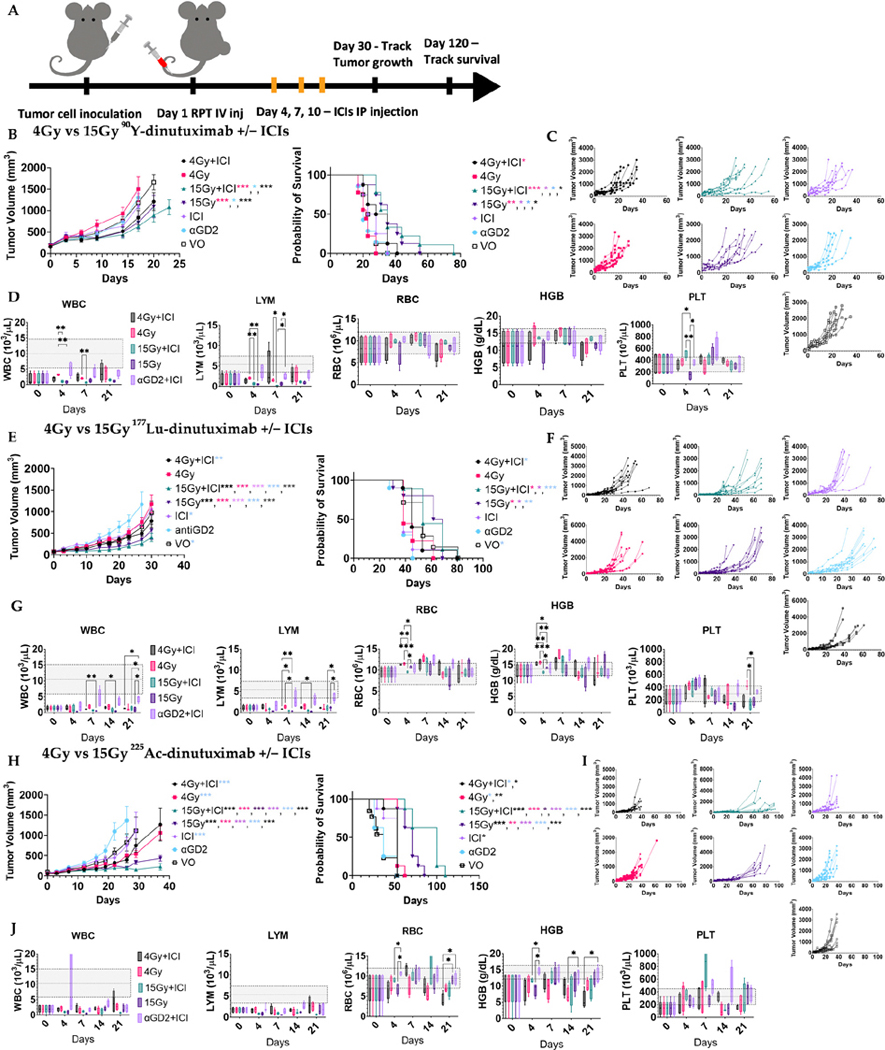
Therapeutic interaction between RPT + ICIs is dose-dependent and radionuclide-dependent. (**A**) Schematic of treatment schedule. B78 bearing mice (n = 8, 9) were randomized into groups and treated on Day 1 with either ^90^Y-, ^177^Lu-, or ^225^Ac-dinutuximab, unlabeled dinutuximab (aGD2) only, ICI only or saline (VO) with radiolabeled doses selected to deliver either 4 Gy (low dose) or 15 Gy (high dose) based on tumor-specific absorbed dose calculations. Anti-PD-L1 and anti-CTLA-4 immune checkpoint inhibitors (ICIs) were administered intraperitoneally on Days 4, 7, and 10 in applicable groups. (**B**,**C**) Effects of ^90^Y-dinutuximab dose on tumor growth and overall survival. (**D**) Complete blood counts on days 0, 4, 7, 21. WBC: white blood cells, LYM: lymphocytes, PLT: platelets, HGB: hemoglobin, RBC: red blood cells. Gray box indicates normal range of respective values in mice. (**E**,**F**) Effects of ^177^Lu-dinutuximab dose on tumor growth and overall survival. (**G**) Complete blood counts on days 0, 4, 7, 14, 21. (**H**,**I**) Effects of ^225^Ac-dinutuximab dose on tumor growth and overall survival. (**J**) Complete blood counts on days 0, 4, 7, 14, 21.

To evaluate the impact of absorbed dose on RPT–ICI synergy, mice received ^90^Y-, ^177^Lu-, or ^225^Ac-dinutuximab at doses corresponding to a low (4 Gy) or high (15 Gy) tumor absorbed dose. Injected activities to achieve these prescriptions were 1.369 MBq and 5.217 MBq for ^90^Y-dinutuximab, 2.22 MBq and 8.251 MBq for ^177^Lu-dinutuximab, and 1.702 kBq and 6.29 kBq for ^225^Ac-dinutuximab, respectively. These were administered with or without dual ICI therapy (anti-CTLA-4 and anti-PD-L1) on days 4, 7, and 10 (Figure 3). Control groups included saline and non-radiolabeled dinutuximab matched to the protein dose used in RPT groups. For both ^90^Y- and ^177^Lu-dinutuximab, 15 Gy groups with or without ICIs significantly improved survival (Figure 3B,C,E,F). ^225^Ac-dinutuximab led to survival improvement across all treatment arms, but the greatest benefit was observed in the 15 Gy + ICI group (Figure 3H,I). There was limited acute toxicity in both doses in all treatment groups (Figures 3D,G,J and S2).

### 3.4. ^225^Ac-Dinutuximab Induces Sustained Type I Interferon Responses

**Figure 4.**
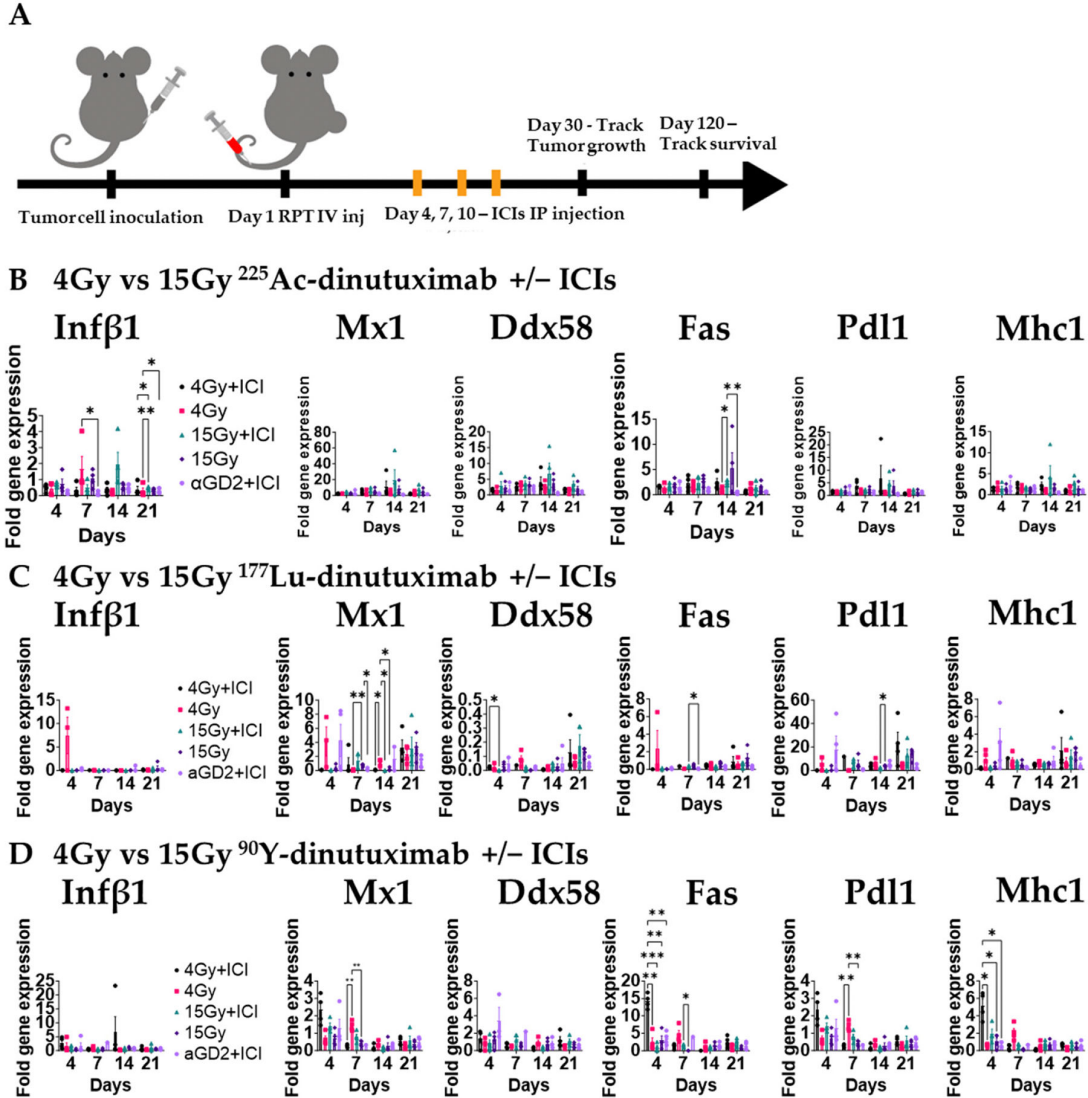
In vivo type I IFN response is activated with ^225^Ac-dinutuximab as compared to ^90^Y- or ^177^Lu-dinutuximab. (**A**) Experimental timeline for gene expression analysis. B78 bearing mice with bilateral flank tumors were treated on Day 1 with either ^90^Y-, ^177^Lu-, or ^225^Ac-dinutuximab, or unlabeled dinutuximab. Mice receiving immune checkpoint inhibitors (ICIs; anti-PD-L1 and anti-CTLA-4) were dosed on Days 4, 7, and 10. Tumors were harvested on Days 4, 7, 14, and 21 (n = 4/timepoint) for gene expression profiling. (**B**) In vivo qPCR results following ^225^Ac-dinutuximab. qPCR was used to quantify gene expression and is reported as fold changed normalized to untreated controls (Day 0, n = 5) performed in duplicates. Two-way ANOVA with Tukey’s HSD post hoc test was used to compare fold change in expression between treatment groups. (**C**) In vivo qPCR results following ^177^Lu-dinutuximab. (**D**) In vivo qPCR results following ^90^Y-dinutuximab.

We next examined how different radionuclides affect the in vivo type I interferon (IFN-I) response. Mice bearing bilateral B78 tumors were treated with ^90^Y-, ^177^Lu-, or ^225^Ac-dinutuximab and ICIs as described above (Figure 4A). Tumors were harvested at days 4, 7, 14, and 21 for qPCR analysis. ^225^Ac-dinutuximab elicited higher and more sustained expression of *Ifnb1* and *Fas* at later timepoints (Figure 4B), suggesting prolonged immunostimulatory activity. In contrast, ^90^Y-dinutuximab induced earlier peaks in *Mx1*, *Fas*, *Pdl1*, and *Mhc1* expression and ^177^Lu-dinutuximab exhibited only moderate upregulation of *Mx1*, *Pdl1*, and *Mhc1* (Figure 4C,D). The prolonged IFN-I activation observed in the 15 Gy ^225^Ac-dinutuximab plus ICI group may contribute to the favorable immunologic milieu for dendritic cell recruitment and effective checkpoint blockade [54,55] (Figure S6H).

### 3.5. 15 Gy ^90^Y- and ^225^Ac-Dinutuximab Enhance CD8^+^ T Cell Infiltration and CD8^+^/Treg Ratio

**Figure 5.**
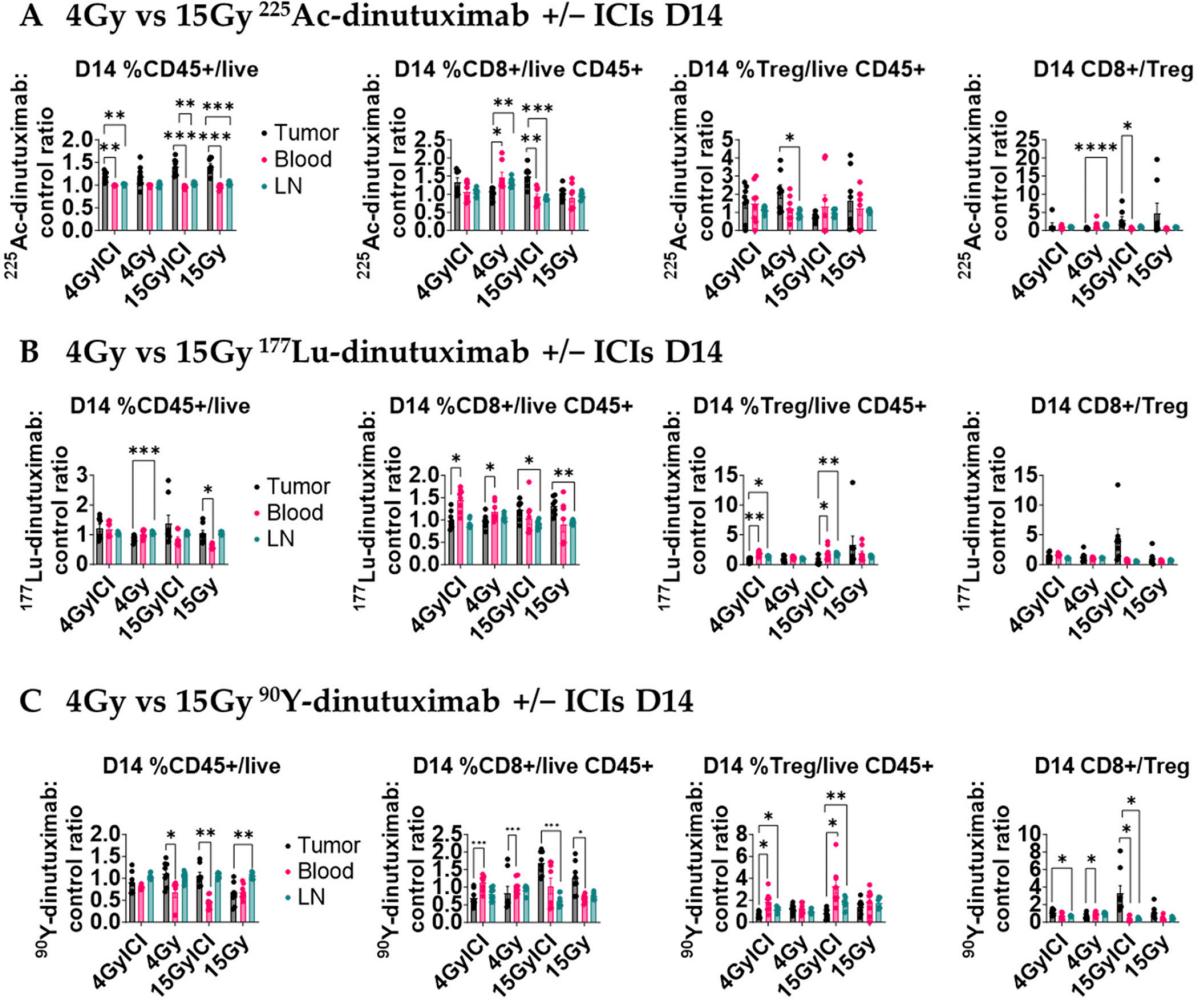
15 Gy of ^90^Y- or ^225^Ac-dinutuximab potentiates immune response when combined with ICIs. (**A**–**C**) Quantification of tumor-infiltrating and systemic immune populations on Day 14 following RPT ± ICI treatment. All mice treatment groups are the same as Figure 4. Mice were euthanized and tumor tissue, blood, and draining inguinal lymph nodes were collected for flow cytometric analysis. Cell populations were normalized to those from mice treated with unlabeled dinutuximab + ICIs and performed in duplicates. Two-way ANOVA with Tukey’s HSD post hoc test was used to compare treatment groups. (**A**) Flow cytometry data in the tumor microenvironment for ^225^Ac-dinutuximab treatment groups. (**B**) Flow cytometry data in the tumor microenvironment for ^177^Lu-dinutuximab treatment groups. (**C**) Flow cytometry data in the tumor microenvironment for ^90^Y-dinutuximab treatment groups.

Given the role of IFN-I in priming CD8^+^ T cell responses [54], we investigated the downstream effects of IFN-1 signaling on the tumor infiltrating lymphocyte (TIL) population following RPT. Using the same B78-bearing mice as for the qPCR experiments above, we investigated the immune cell composition of host immune organs (blood and tumor-draining inguinal lymph node) and disaggregated tumor tissue compared to control mice by flow cytometry. All data were normalized to the control mice treated with unlabeled dinutuximab combined with ICIs. The day 14 CD45^+^, CD8^+^, regulatory T cell (Treg), and CD8^+^/Treg ratio are reported for all of the tissues following injection on day 1 (Figure 5). In Figures S4–S6, CD45^+^, CD8^+^, regulatory T cell (Treg), CD8^+^/Treg ratio, %PD1^+^ CD8^+^ T cells, CD4^+^ T cells, NK cells, CD11b^+^ cells, M1- and M2-like macrophages, and dendritic cells are reported at days 3, 7, 14, and 21 are reported for same treatment groups and tissues. The CD8^+^/Treg ratio significantly increased (*p* > 0.05) in tumor microenvironment relative to other tissues with 15 Gy + ICI ^225^Ac- and ^90^Y-dinutuximab groups (Figure 5A,C). Notably, a significant change in CD8^+^ frequency was observed in the tumor for these groups (^225^Ac-dinutuximab tumor: blood, *p* < 0.005; lymph node, *p* < 0.001 and ^90^Y-dinutuximab tumor: lymph node, *p* < 0.0001). This finding suggests that the limited tissue penetration capacity of the monoclonal antibody may stimulate the composition of immune cells in the tumor microenvironment at high-dose compared to the low-dose small-molecule RPT [4,7,8]. In addition, 15 Gy ^225^Ac-dinutuximab with ICIs significantly increased the frequency of dendritic cells in the tumor microenvironment relative to the tumor draining lymph node (*p* < 0.005) on D21 correlative to IFN-1 activation (Figure S6H).

## 4. Discussion

In this study, we present in vivo quantitative imaging and dosimetry data for ^90^Y-, ^177^Lu-, and ^225^Ac-labeled dinutuximab in syngeneic melanoma (B16/B78) and neuroblastoma (9464D/9464D:GD2^+^) models. In the melanoma model, survival was significantly improved with absorbed dose-matched 15 Gy administration of all three RPTs. However, only ^225^Ac-dinutuximab demonstrated a significant additive survival benefit when combined with ICIs. Our immunophenotyping and gene expression data further correlated these survival trends with distinct longitudinal changes in tumor and systemic immune cell composition.

The cooperative therapeutic interaction between RPT and ICIs is critically dependent on both the delivery mechanism and microdosimetric distribution. Antibody-based RPT exploits antigen specificity to preferentially deliver ionizing radiation to malignant tissues while minimizing off-target toxicity. In our case, dinutuximab’s affinity for GD2 [15,17] facilitated selective tumor uptake across all conjugated radionuclides. Nevertheless, the restricted tissue penetration of full-length antibodies, coupled with the inherently short range of α-particles, results in a more pronounced peak-to-valley radiation distribution within tumors compared with β-particle emitters (Figure S7). This dose heterogeneity is known to influence therapeutic outcomes [56] and may explain the superior results observed with 15 Gy of ^225^Ac-dinutuximab combined with ICIs. Importantly, emerging evidence suggests that such heterogeneity may not be purely detrimental. As shown in previous brachytherapy studies [57], dose distributions that preserve low-dose regions within the tumor microenvironment (TME) may be critical for sustaining immune activity, particularly dendritic cell function and migration. In line with this, Patel et al. demonstrated that in the B78 melanoma model treated with the small molecule RPT NM600, immune stimulation was optimal at ~2 Gy but diminished at 5 Gy, underscoring the importance of low-dose regions for productive antitumor immunity [4].

Although α-particles have a limited range (<100 μm) in tissues, their high linear energy transfer (LET) enables them to induce dense, irreparable DNA double-strand breaks, which are highly immunogenic [58]. In our experiments, ^90^Y-dinutuximab treatment led to early upregulation of antigen presentation (*Mhc1*, *Pdl1*) and apoptotic markers (*Fas*). In contrast, ^225^Ac-dinutuximab produced a more prolonged IFN- I response, a critical mediator of antitumor immunity through its enhancement of dendritic cell activation and CD8^+^ T cell infiltration [59,60]. This was corroborated by flow cytometry, which demonstrated that the 15 Gy ^225^Ac-dinutuximab + ICI group showed the highest CD8^+^/Treg ratio and dendritic cell recruitment within the tumor microenvironment by day 21, consistent with the strongest observed survival benefit. However, ^177^Lu-dinutuximab did not demonstrate the same degree of early upregulation observed with ^90^Y-dinutuximab, despite both radionuclides being β-emitters. This disparity may be attributable to the lower maximum β energy and substantially shorter tissue penetration of ^177^Lu (0.5 MeV; ~1.5–2 mm) relative to ^90^Y (2.28 MeV; ~11 mm) [61], which results in more spatially confined tumor cell injury and may consequently attenuate or delay immunomodulatory signaling without achieving the effective dose heterogeneity observed with ^225^Ac (Figure S7). Furthermore, previous reports indicate that ^90^Y induces broader tumor and stromal disruption, facilitating the release of tumor-associated antigens and danger-associated molecular patterns, thereby promoting more rapid and pronounced immune activation [62,63].

Our results strongly suggest that administered activity alone (e.g., in MBq or μCi) is an inadequate predictor of therapeutic outcome, particularly when the goal is to stimulate an immune response. Tumor-specific factors such as antigen density, vascularity, and microenvironmental composition significantly influence the actual absorbed dose. Our voxel-based Monte Carlo dosimetry revealed considerable variability in tumor absorbed dose between models and radionuclides, despite equal injected activities. These findings reinforce the necessity of individualized dosimetry to guide therapeutic decision-making and accurately interpret treatment outcomes. However, implementing patient-specific dosimetry in the clinical setting presents several challenges. Accurate dosimetry requires multiple timepoint imaging sessions—typically via PET or SPECT—over a span of several days. While feasible in preclinical models, this approach imposes substantial logistical, financial, and radiation exposure burdens in human patients [50]. Multiple imaging appointments, prolonged scanner occupancy, and radiopharmaceutical accessibility across timepoints pose barriers in many clinical environments. These are compounded by vulnerable populations, including pediatric, elderly, or terminally ill patients. As a result, clinical protocols often default to empiric or fixed dosing regimens, which risk under- or over-treating individuals. To address this gap, there is an urgent need for streamlined and clinically viable dosimetry methods—such as single-timepoint dose estimation, machine learning-based modeling, or surrogate imaging biomarkers [64,65]. These strategies could reduce the patient burden while maintaining sufficient precision for dose optimization.

We acknowledge the limitations of using murine models, which do not fully recapitulate the complexity of human tumors or immune systems [66]. While these models are indispensable for mechanistic and early translational studies, further validation in large-animal models and clinical trials will be necessary to confirm the therapeutic implications of our findings. Another limitation is that despite their advantages, radiolabeled monoclonal antibodies are underrepresented in clinical RPT protocols compared to small molecules or antibody fragments [67]. This is at least partially attributable to their large size, which restricts tumor penetration and promotes dose heterogeneity [18]. As such, optimizing delivery strategies through alternative formats like minibodies or bispecific antibodies remains a critical area of future research [53,68]. Lastly, although ^225^Ac-dinutuximab demonstrated robust immunostimulatory activity, only a single α-emitting radionuclide was evaluated. As such, it remains premature to conclude that ^225^Ac is categorically superior to β-emitting constructs. Inclusion of additional α-emitters with distinct physical properties—such as ^212^Pb or ^149^Tb—would provide a more comprehensive assessment of α-particle–mediated therapeutic efficacy.

## 5. Conclusions

This study demonstrates that the delivery mechanism and resulting microdosimetry of radiopharmaceutical therapy are central to achieving effective synergy with immune checkpoint inhibitors. Our findings show that functionally distinct immune outcomes can arise even when using the same antibody vector, emphasizing the critical role of radionuclide properties—particularly radiation type, LET, and spatial distribution—in shaping tumor immunogenicity.

We confirm that dinutuximab selectively targets GD2-positive tumors and supports the sustained intratumoral retention of radiolabeled agents. However, the immunological and survival outcomes varied markedly depending on the radionuclide used. The short-range, high-LET emission of ^225^Ac enabled localized immune priming, resulting in the most robust IFN-I responses, CD8^+^ T cell infiltration, and dendritic cell recruitment—key hallmarks of successful immunotherapy. In contrast, β-emitters like ^90^Y and ^177^Lu produced less pronounced or more transient effects in our models, despite equivalent injected activities.

Our voxel-based dosimetry analysis revealed that injected activity does not correlate directly with tumor-absorbed dose, underscoring the inadequacy of current empirical dosing paradigms. Instead, personalized dosimetry is necessary to optimize immunological outcomes and therapeutic efficacy. This biologically guided approach enables a more accurate interpretation of treatment responses and the design of more effective combination protocols.

We suggest that for combined modality treatments with RPT and immunotherapies, RPT microdosimetry is a biological imperative. Sub-therapeutic or poorly distributed radiation may not only fail to activate immune pathways but may also contribute to immune suppression or resistance. Because antibody-based RPT is limited by tissue penetration and spatial heterogeneity, understanding different tumor heterogeneity is crucial for maximizing therapeutic benefit—particularly in combination with immunomodulators.

Together, these results advocate for a paradigm shift from standardized dosing to biologically informed, individualized RPT planning. Integrating personalized microdosimetry into both preclinical and clinical studies will be essential for realizing the full potential of RPT and ICI combinations in precision oncology.

## Supplementary Material

Supplementary Material

The following supporting information can be downloaded at https://www.mdpi.com/article/10.3390/radiation5040039/s1: Figure S1: Biodistribution of ^89^Zr-dinutuximab in melanoma and neuroblastoma murine models; Figure S2: Statistical tests performed on survival and tumor growth curves; Figure S3: Acute toxicity profile of ^177^Lu and ^225^Ac-dinutuximab; Figure S4: ^90^Y-dinutuximab tumor immune cell composition in tumor microenvironment; Figure S5: ^177^Lu-dinutuximab tumor immune cell composition in tumor microenvironment; Figure S6: ^225^Ac-dinutuximab tumor immune cell composition in tumor microenvironment; FigureS7: Activity map distribution of sectional slices of tumor treated with ^90^Y-, ^177^Lu-, and ^225^Ac-dinutuximab from Figure 1G; Table S1: List of TaqMan probes utilized for quantitative RT-PCR experiments; Table S2: List of flow cytometry antibody targets, clones, and fluorophores.

## Figures and Tables

**Table 1.** Injected activity of RPT for tumor absorbed dose prescriptions.

RPT	Tumor Absorbed Dose Prescription (Gy)	Injected Activity
^90^Y-dinutuximab	4	1.369 MBq
^90^Y-dinutuximab	15	5.217 MBq
^177^Lu-dinutuximab	4	2.22 MBq
^177^Lu-dinutuximab	15	8.251 MBq
^225^Ac-dinutuximab	4	1.702 kBq
^225^Ac-dinutuximab	15	6.29 kBq

## Data Availability

The data that support the findings of this study are available from the corresponding author upon reasonable request.

## Abbreviations

The following abbreviations are used in this manuscript:

^225^Ac: Actinium-225
^89^Zr: Zirconium-89
^90^Y: Yttrium-90
^177^Lu: Lutetium-177
Ab: Antibody
B78: Murine melanoma cell line
CD4: Cluster of Differentiation 4 (helper T cell marker)
CD8: Cluster of Differentiation 8 (cytotoxic T cell marker)
CD11b: Cluster of Differentiation 11b (myeloid cell marker)
CT: Computed Tomography
CTLA-4: Cytotoxic T-Lymphocyte Antigen 4
DFO: Desferrioxamine
DTPA: Diethylenetriaminepentaacetic Acid
Fas: Tumor Necrosis Factor Receptor Superfamily Member 6 (apoptosis marker)
GD2: Disialoganglioside 2
Gy: Gray (unit of absorbed dose)
γH2AX: Phosphorylated Histone H2AX (marker of DNA damage)
ICI: Immune Checkpoint Inhibitor
IFN: Interferon
kBq: Kilobecquerel
MBq: Megabecquerel
MHC I: Major Histocompatibility Complex Class I
mAb: Monoclonal Antibody
MX1: Myxovirus Resistance Protein 1 (Type I IFN response marker)
NK: Natural Killer (cell)
PBS: Phosphate-Buffered Saline
PD-L1: Programmed Death-Ligand 1
PET: Positron Emission Tomography
qPCR: Quantitative Polymerase Chain Reaction
RPT: Radiopharmaceutical Therapy
SPECT: Single Photon Emission Computed Tomography
TME: Tumor Microenvironment
Treg: Regulatory T Cell
