# Supplementary material for: Radionuclide-Dependent Stimulation of Antitumor Immunity in GD2-Targeted Radiopharmaceutical Therapy Combined with Immune Checkpoint Inhibitors
Source: Radiation (Basel). Author manuscript; Available in PMC 2026 Apr 22. (PMC13098876; doi:10.3390/radiation5040039)
Supplement: Supplementary Material [file NIHMS2129285-supplement-Supplementary_Material.pdf]

Supplementary Materials

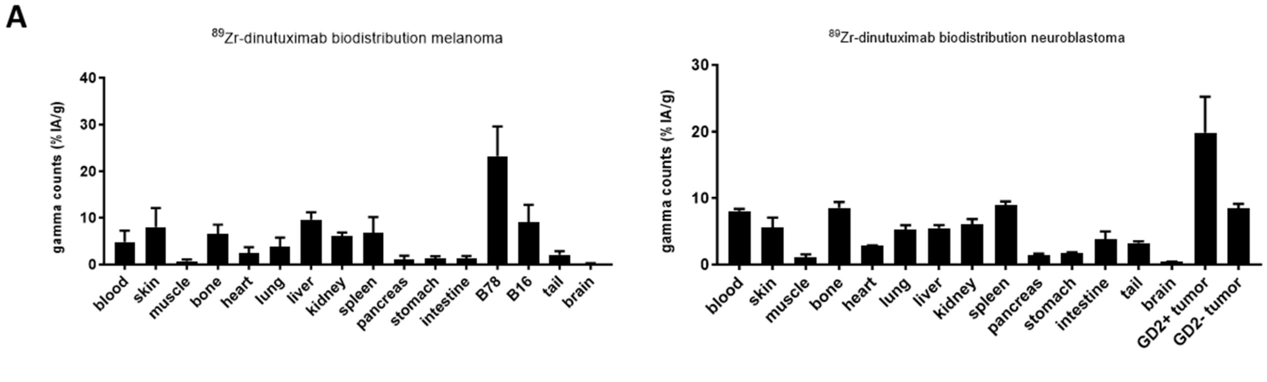

Figure S1. Biodistribution of ^{89}Zr-dinutuximab in melanoma and neuroblastoma murine models

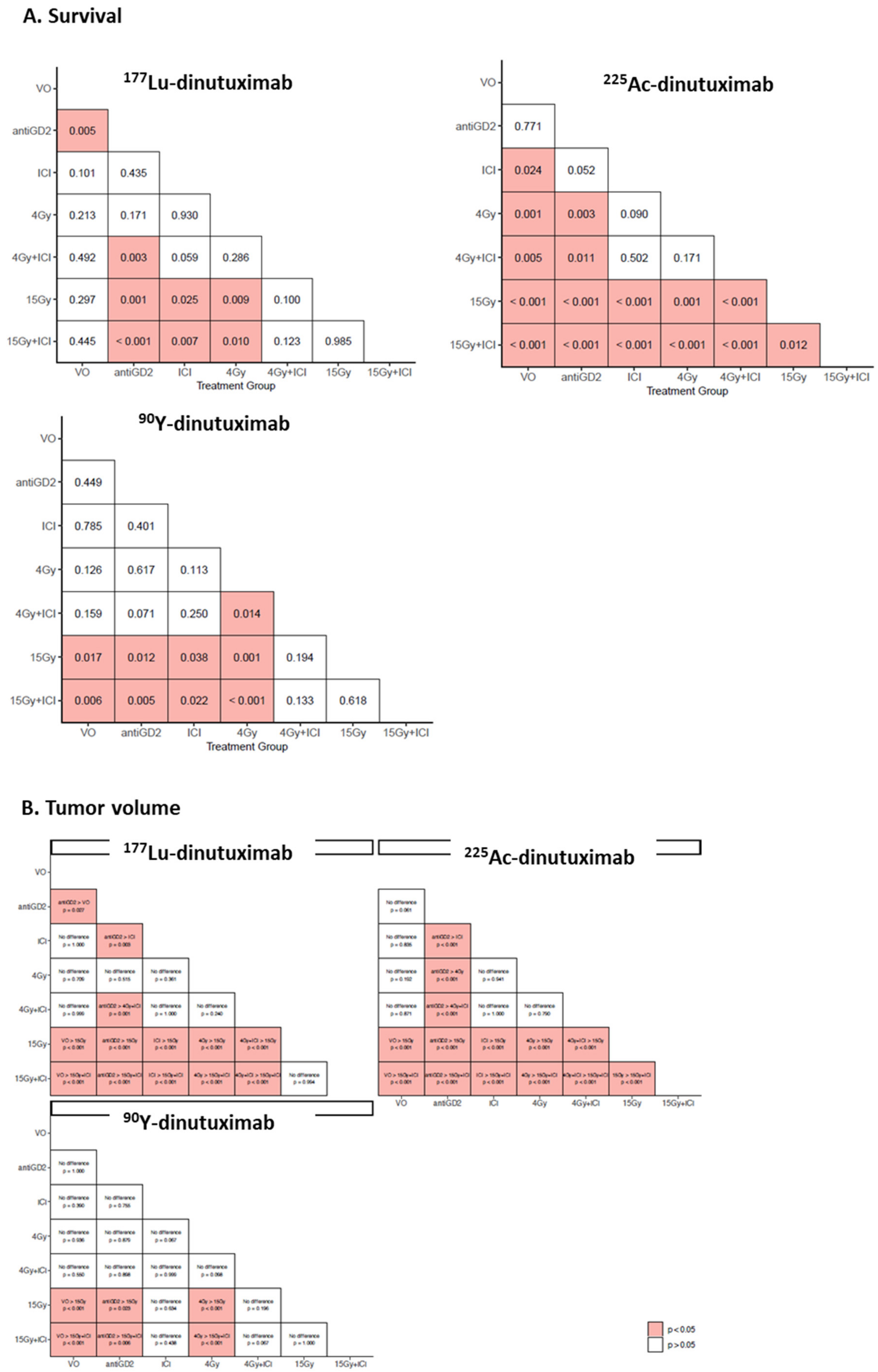

Figure S2. Statistical tests done on survival and tumor growth curves

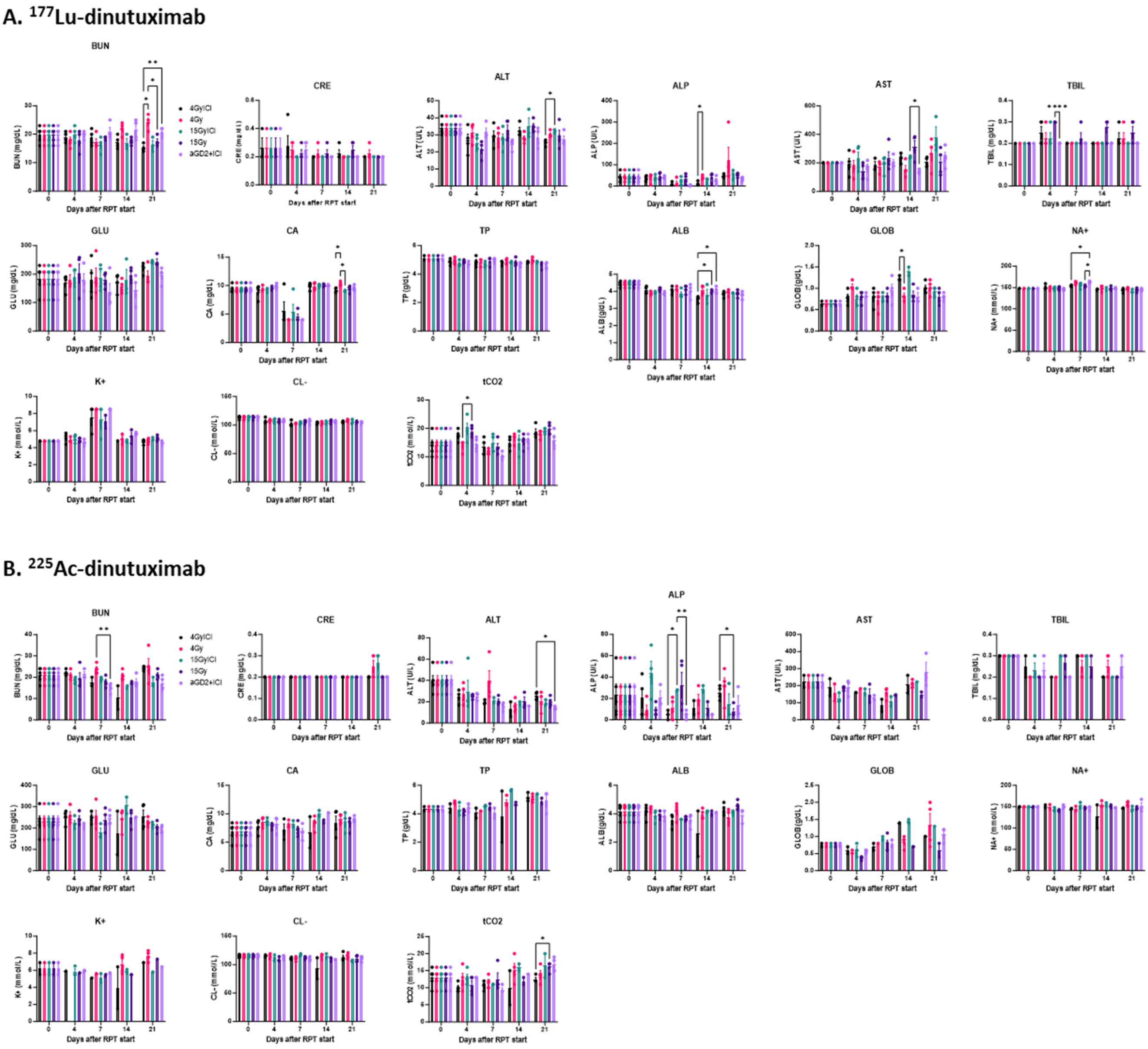

Figure S3. Acute toxicity profile of ^{177}Lu and ^{225}Ac-dinutuximab

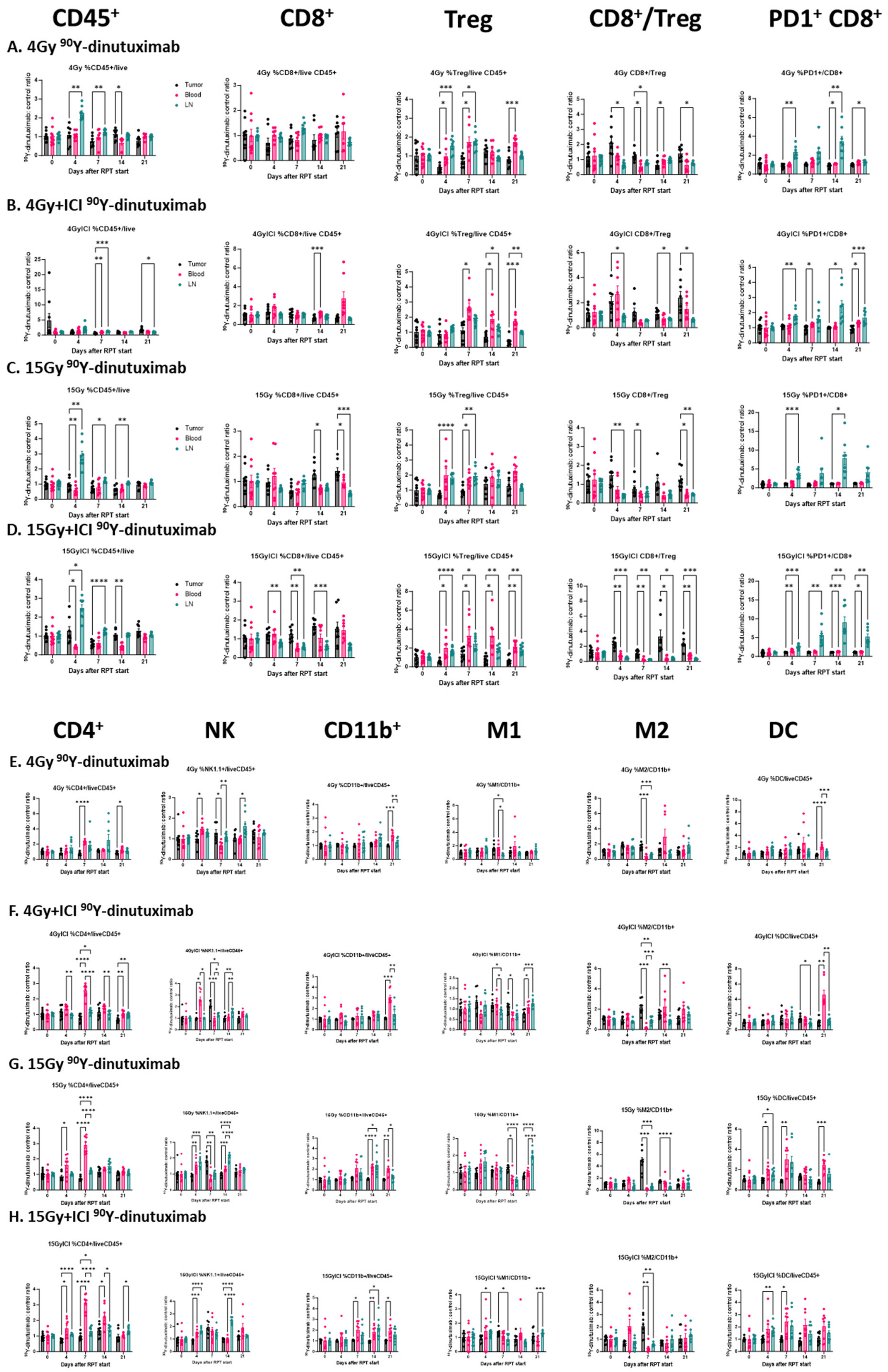

Figure S4. ^{90}Y-dinutuximab tumor immune cell composition in tumor microenvironment

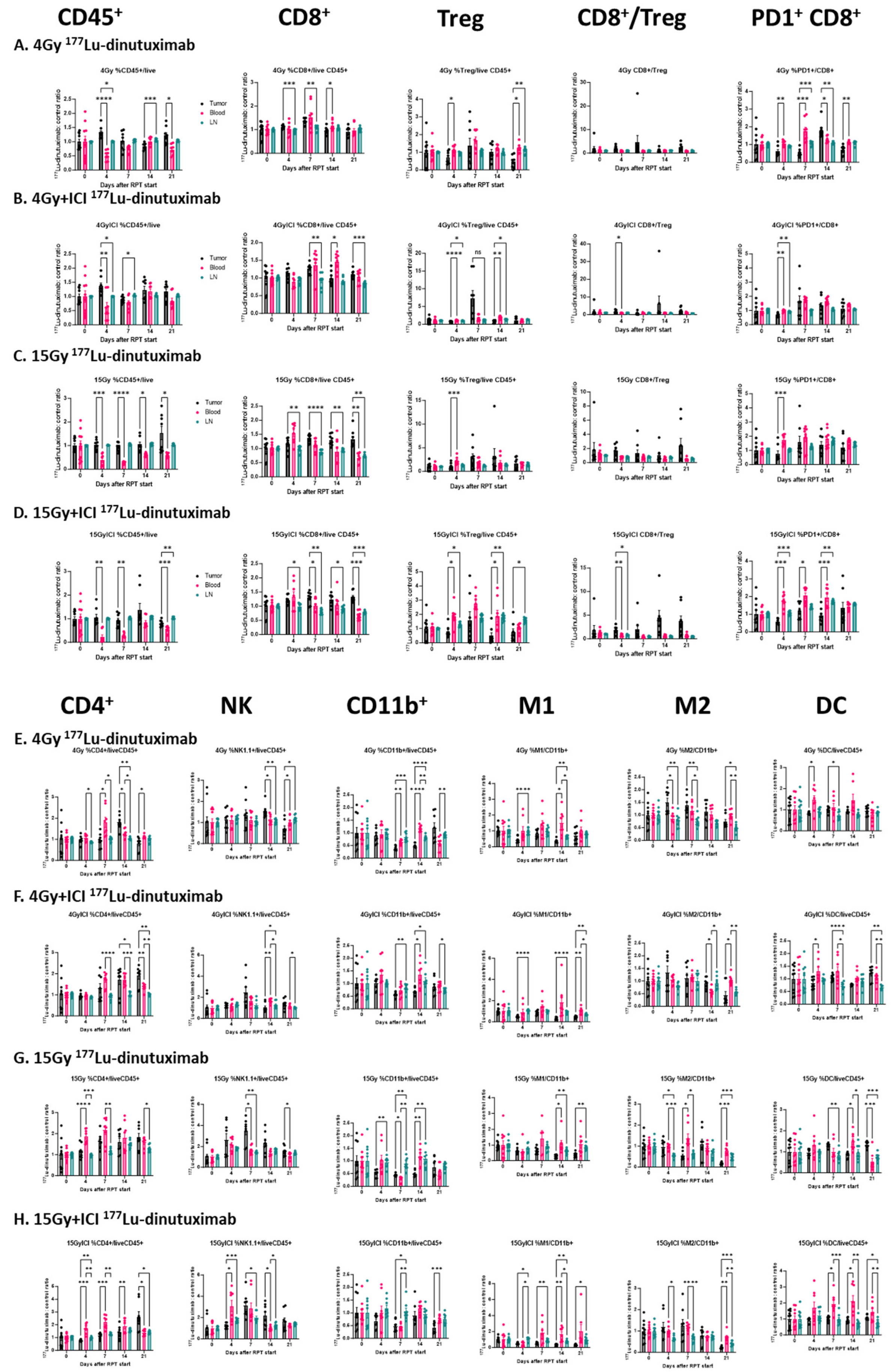

Figure S5. ^{177}Lu-dinutuximab tumor immune cell composition in tumor microenvironment

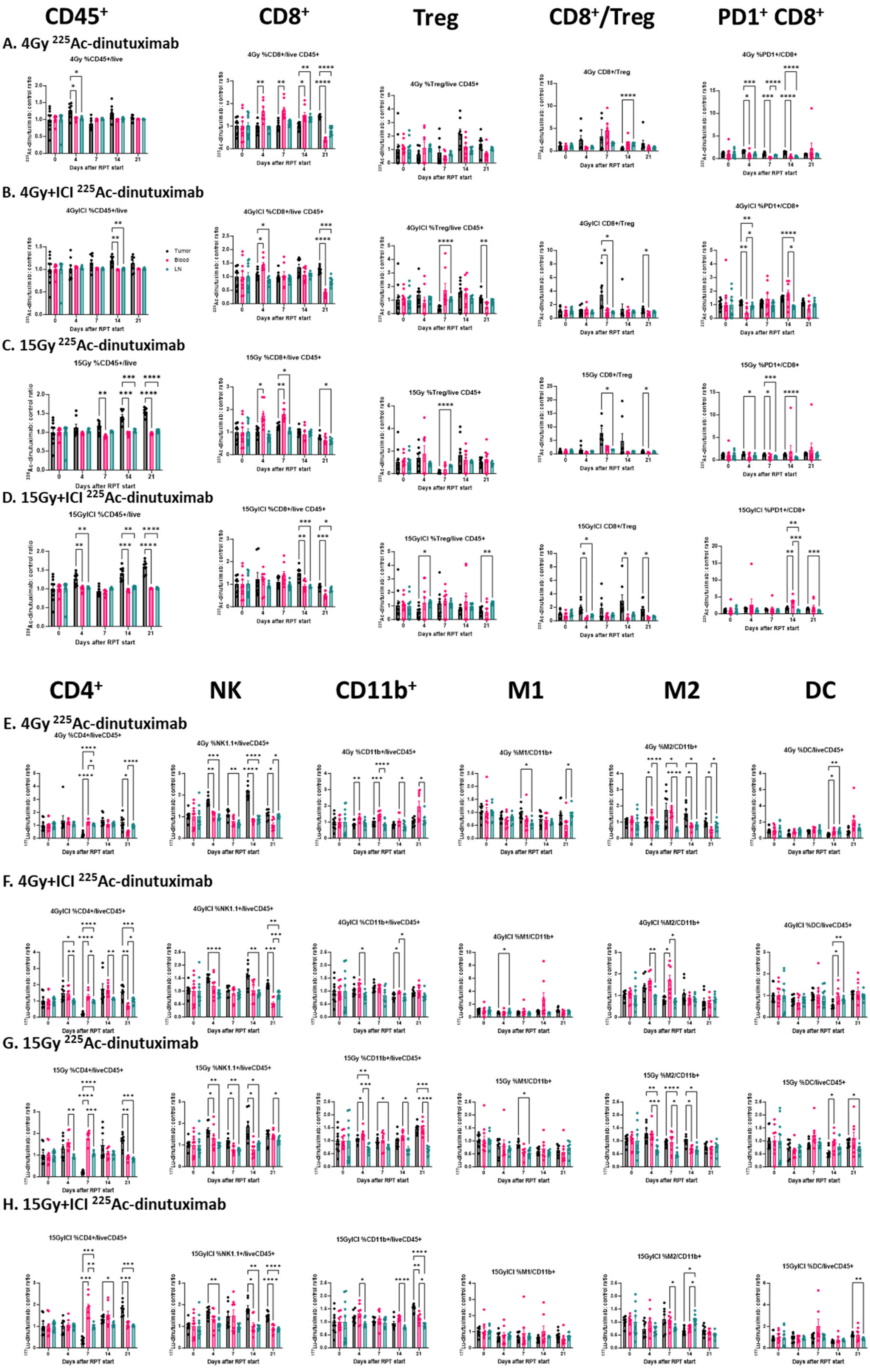

Figure S6. ^{225}Ac-dinutuximab tumor immune cell composition in tumor microenvironment

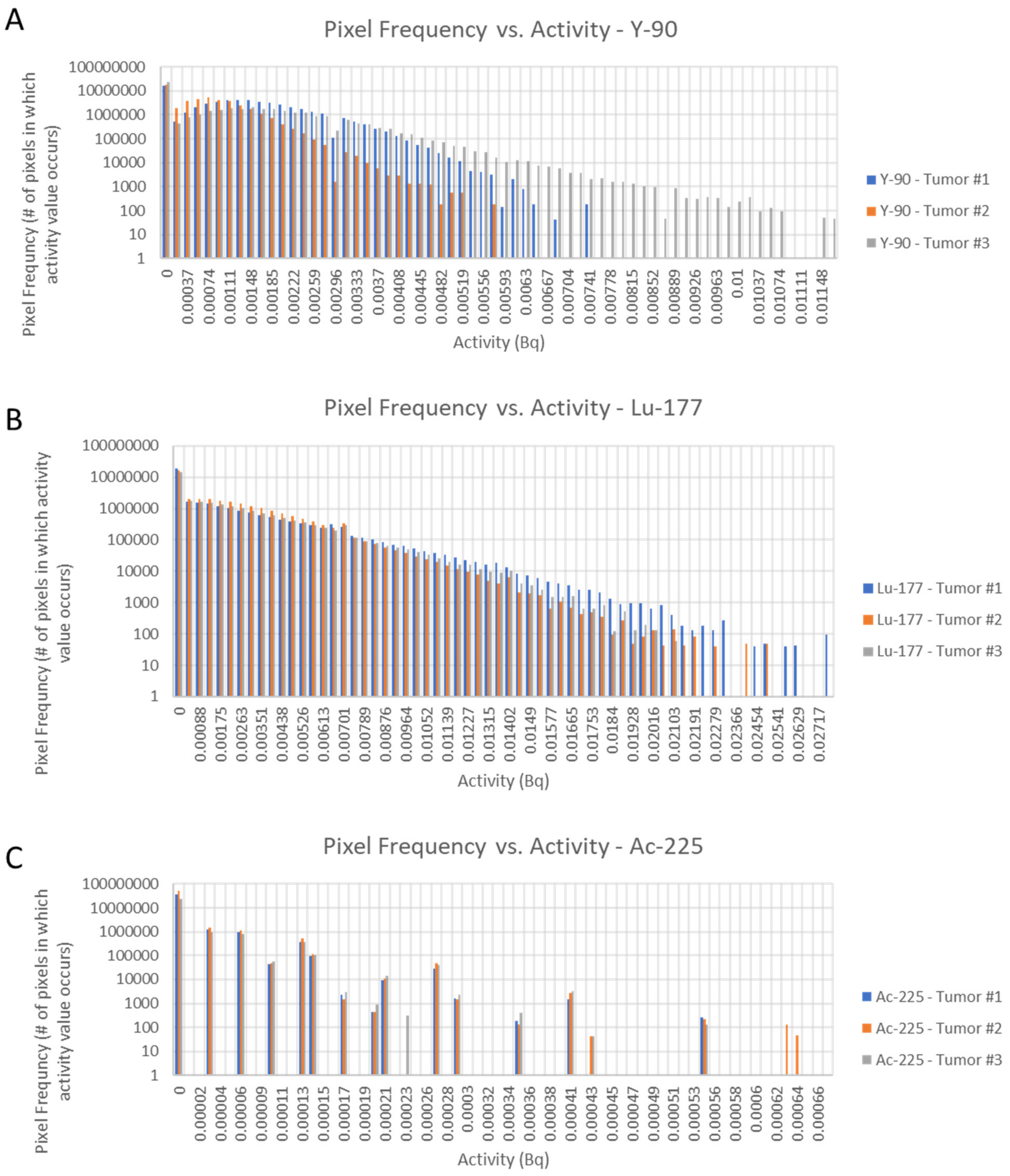

Figure S7. Activity map distribution of sectional slices of tumor treated with ^{90}Y-, ^{177}Lu-, and ^{225}Ac-dinutuximab from Figure 1G.

Table S1. List of TaqMan probes utilized for quantitative RT-PCR experiments

Gene Name	Assay ID
Hprt	Mm03024075_m1
Ifnb1	Mm00439552_s1
Mx1	Mm00487796_m1
Fas	Mm01204974_m1
Pdl1 (CD274)	Mm03048248_m1
Mhc1 (H2-D)	Mm04208017_mH
Ddx58	Mm01216853_m1

Table S2. List of flow cytometry antibody targets, clones, and fluorophores

Name	Clone	Fluorophore	Catalog number
CD4	RM4-5	FITC	BioLegend 100510
CD11c	N418	PerCP-Cy5.5	BioLegend 117328
F4/80	BM8	PE/Dazzle 594	BioLegend 123146
NK1.1	PK136	PE-Cy5	BioLegend 108716
FOXP3	FJK-16s	PE-Cy7	Thermo Scientific 25-5773-82
CD279 (PD-1)	RMP1-30	BV421	BioLegend 109121
I-A/I-E (MHC-II)	M5/114	BV605	BioLegend 107639
CD45	30-F11	BV510	BioLegend 103138
CD11b	M1/70	BV711	BioLegend101242
CD25	PC61	APC	BioLegend 102012
CD8a	53-6.7	Alexa700	BioLegend100730
